# Identification of mitochondrial related signature associated with immune microenvironment in Alzheimer’s disease

**DOI:** 10.1186/s12967-023-04254-9

**Published:** 2023-07-11

**Authors:** Yaodan Zhang, Yuyang Miao, Jin Tan, Fanglian Chen, Ping Lei, Qiang Zhang

**Affiliations:** 1 Department of Geriatrics, Tianjin Medical University General Hospital, Anshan Road No. 154, Tianjin, 300052 China; 2 Tianjin Geriatrics Institute, Anshan Road No. 154, Tianjin, 300052 China; 3 Haihe Laboratory of Cell Ecosystem, Tianjin Medical University General Hospital, Anshan Road No. 154, Tianjin, 300052 China; 4 Tianjin Neurological Institute, Tianjin Medical University General Hospital, Tianjin, China

**Keywords:** Alzheimer's disease, Mitochondria, Immune infiltration, Bioinformatics analysis

## Abstract

**Background:**

Alzheimer's disease (AD) is the most common neurodegenerative disease. Mitochondrial dysfunction and immune responses are important factors in the pathogenesis of AD, but their crosstalk in AD has not been studied. In this study, the independent role and interaction of mitochondria-related genes and immune cell infiltration in AD were investigated using bioinformatics methods.

**Methods:**

The datasets of AD were obtained from NCBI Gene Expression Omnibus (GEO), and the data of mitochondrial genes was from MitoCarta3.0 database. Subsequently, differential expression genes (DEGs) screening and GSEA functional enrichment analysis were performed. The intersection of DEGs and mitochondrial related genes was used to obtain MitoDEGs. The MitoDEGs most relevant to AD were determined by Least absolute shrinkage and selection operator and multiple support vector machine recursive feature elimination, as well as protein–protein interactions (PPI) network and random forest. The infiltration of 28 kinds of immune cells in AD was analyzed by ssGSEA, and the relationship between hub MitoDEGs and the proportion of immune infiltration was studied. The expression levels of hub MitoDEGs were verified in cell models and AD mice, and the role of OPA1 in mitochondrial damage and neuronal apoptosis was investigated.

**Results:**

The functions and pathways of DEGs were significantly enriched in AD, including immune response activation, IL1R pathway, mitochondrial metabolism, oxidative damage response and electron transport chain-oxphos system in mitochondria. Hub MitoDEGs closely related to AD were obtained based on PPI network, random forest and two machine learning algorithms. Five hub MitoDEGs associated with neurological disorders were identified by biological function examination. The hub MitoDEGs were found to be correlated with memory B cell, effector memory CD8 T cell, activated dendritic cell, natural killer T cell, type 17 T helper cell, Neutrophil, MDSC, plasmacytoid dendritic cell. These genes can also be used to predict the risk of AD and have good diagnostic efficacy. In addition, the mRNA expression levels of BDH1, TRAP1, OPA1, DLD in cell models and AD mice were consistent with the results of bioinformatics analysis, and expression levels of SPG7 showed a downward trend. Meanwhile, OPA1 overexpression alleviated mitochondrial damage and neuronal apoptosis induced by Aβ1-42.

**Conclusions:**

Five potential hub MitoDEGs most associated with AD were identified. Their interaction with immune microenvironment may play a crucial role in the occurrence and prognosis of AD, which provides a new insight for studying the potential pathogenesis of AD and exploring new targets.

**Supplementary Information:**

The online version contains supplementary material available at 10.1186/s12967-023-04254-9.

## Introduction

Alzheimer's disease (AD) is a progressive neurodegenerative disease and the main cause of dementia, accounting for 60–80% of dementia patients. Dementia refers to the decline of many brain functions, including memory, reasoning and language. The progression of AD can last 15–25 years. AD is mainly characterized by memory loss and cognitive impairment, and the patient's ability to independently carry out daily activities is reduced. The biggest risk factors for AD are advanced age (over 65 years) and carrying at the apolipoprotein E ε4 (APOE ε4) allele [1, 2]. New biomarkers including PET scans and plasma measurements of amyloid β (Aβ) and phosphorylated tau (p-tau) hold great help for AD diagnosis. In addition to the biochemical amyloid and tau pathology that are core features of AD, microglia responses, the vascular system, blood–brain barrier, the peripheral immune system, glymphatic and other clearance systems, and potentially the gastrointestinal microbiome influence the clinical progression of the disease [2]. Current treatments for AD include cognitive improvement therapy, treatment of neuropsychiatric symptoms, and disease modification therapy. But some drugs are still being studied and are not very effective. Therefore, new progress in the diagnosis and treatment of AD remains an urgent task.

It is widely believed that neuroinflammation in AD is mediated by microglia and astrocytes [3]. Substantial evidence has emerged for the involvement of innate and adaptive immune responses in the development or progression of AD [4, 5]. More recently, it has been found that increased T cell infiltration promotes crosstalk between T cells and microglia, leading to further acceleration of neuroinflammation [6, 7]. Similarly, peripheral B lymphocytes can enter the central nervous system of AD patients, break through the blood–brain barrier, and promote the activation of immune response by interacting with the stationed brain cells [8]. Phenotypic changes of circulating neutrophils at different stages of AD affect systemic chronic inflammation and the rate of cognitive decline [9]. Natural killer cells, peripheral dendritic cells, and mast cells were all associated with an increased risk of AD injury [10, 11]. These findings highlight the critical role of immune cells in AD.

Mitochondria are indispensable organelles to maintain cell energy metabolism and signal organelles to maintain cell biological functions. Compounds that reduce ROS levels, regulate mitochondrial metabolism, enhance mitochondrial biogenesis may be potential methods to delay aging and treat neurodegenerative diseases by restoring mitochondrial homeostasis [12, 13]. Auwerx et al. have shown that amyloid-β proteotoxic could be reduced by increasing mitochondrial proteostasis [13]. In addition, Fang et al. found that impairment of mitophagy induces cognitive deficits by affecting Aβ and Tau accumulation through increased oxidative damage and mitochondrial energy defects [14]. Meanwhile, mitochondrial miRNAs have also been shown to affect ATP production, oxidative stress, mitochondrial dynamics, and thus regulate AD progression [15]. It must be mentioned that mitochondria dysfunction plays an important role in exacerbating inflammatory responses in multiple ways [16]. Dysfunctional mitochondria release mitochondrial components [including mitochondrial DNA (mtDNA)] through a variety of mechanism that induce inflammatory responses through pattern recognition receptors (PRRs). For example, impaired mitophagy contributes to inflammation, and likewise, an increase in mitochondrial ROS and mtDNA stimulates the cyclic GMP-AMP synthase (cGAS)—stimulator of interferon genes (STING) pathway to increase interferon signaling [17, 18]. In ageing microglia, the reduced glucose flux and mitochondrial respiration lead to maladaptive proinflammatory responses [19]. Drugs to prevent AD by improving mitochondrial function has been widely studied, such as mitochondrial fission protein inhibitors, drugs that promote fusion, as well as antioxidants including MitoQ, vitamin E and curcumin [20, 21]. The mitochondrial gatekeeper protein, VDAC1, has been shown to be a promising drug candidate for AD [22]. However, the crosstalk between mitochondrial genes and the immune microenvironment has been little studied in AD.

Based on the important role of mitochondria in the occurrence and development of AD, we will further understand the role of mitochondria-related genes in regulating mitochondrial function and immune progression, so as to provide certain reference value for the pathogenesis and diagnosis of AD. We explored the potential molecular mechanism by searching GEO database, and analyzed the role of mitochondria-related genes in AD development and their relationship with immune infiltration, which contributed to a better understanding of the immune metabolism in the development of AD.

## Materials and methods

### Data acquisition

The series matrix files and platform’s annotation files were obtained from the NCBI GEO public database. GSE122063 contained RNA expression data generated by the GPL16699 platform, including 56 AD samples and 44 non-demented (ND) controls from humans. The data were background corrected, normalized between arrays, and log2 transformed [23]. Samples from three datasets GSE132903, GSE33000 and GSE44770 were selected to validate the hub genes. GSE132903 is annotated by GPL10558 and includes 97 AD samples and 98 ND samples from humans. GSE44770 is annotated by GPL4372, comprising 129 AD samples and 101 ND samples from humans. GSE33000 is annotated by GPL4372 and comprises 310 AD samples and 157 ND samples.

### Differential expression genes (DEGs) analysis

Differential expression genes of expression series matrix was identified using the “limma” R package in R software [24]. And principal component analysis (PCA) was obtained with R package “factoextra” [25]. Genes with adj.p.val (false discovery rate (FDR)-adjusted) < 0.05 and |log2 (Fold-change)|> 0.58 (Fold change > 1.5) [26, 27] were identified as DEGs. The “pheatmap” [28] and “ggplot2” [29] packages were used to visualize the DEGs results, creating heat maps and volcano plots.

### Functional enrichment analysis of gene

Gene Set Enrichment Analysis (GSEA) [30] was performed using R package “clusterProfiler” [31] and “GSEABase” [32]. The Molecular Signatures Database (MSigDB) “c2.all.v2023.1.Hs.entrez” and “c5.all.v2023.1.Hs.entrez” as the reference gene set. The results were visualized with R package “enrichplot” [33].

### Extraction of mitochondria‑related DEGs (MitoDEGs)

There are 1136 mitochondria-related genes in the MitoCarta3.0 database [34]. MitoDEGs were obtained by crossing the above DEGs with mitochondria‑related genes, which were visualized via Venn diagram [35]. Then, the top 50 MitoDEGs with the most significant differences were visualized using R packets “pheatmap” [28].

### Identification of hub MitoDEGs

Based on the MitoDEGs obtained from the above analysis, the “glmnet” package [36] was used to identify hub MitoDEGs by performing the Least Absolute shrinkage and selection operator (LASSO) logistic regression. The specific parameters: family = “binomial”, nfolds = 10, and tenfold cross-validation was used to adjust the optimal value of the parameter λ. The minimum lambda was defined as the optimal value. In this way, more accurate prediction models can be obtained by this method.

Support vector machine (SVM) is a powerful binary classifier which establishes a classification hyperplane as a decision surface. SVM-RFE (recursive feature elimination) was used to optimize the prediction model by reducing the eigenvectors generated by SVM, using the “1071” R package [37] with the specific parameters: halfve. above = 20 and k = 10. And tenfold cross-validation was used to make the algorithm more accurate. The feature MitoDEGs are obtained by overlapping the results of the LASSO and SVM-RFE.

### Analysis of protein–protein interactions

PPI networks were obtained with the STRING database and visualized using Cytoscape 3.9.1 [38]. The plug-ins CytoHubba and MCODE provided by Cytoscape were used to obtain key MitoDEGs.

### Random forests (RF) to screen hub genes

Random forest is an algorithm of recursive partition based on the construction of binary tree. We screened the hub genes with the R package “randomForest” [39] with the following parameters: ntree = 500, mtry = 3, importance = T, and the Gini index was used as an important measure. Random forest algorithm was used to sort the DEGs according to the decrease in Gini index, and the top six genes with significant values greater than 3 were selected for downstream analysis.

### Immune infiltration analysis

To better identify the characteristics of immune cells in the brain tissue of normal population and AD patients, we conducted a single-sample gene-set enrichment analysis (ssGSEA) algorithm to estimate the differential composition of infiltration abundance of 28 immune cell types between the two groups [40] based on gene expression profiles from microarray. The correlation between the expression of hub MitoDEGs and the distribution of immune cells was revealed by spearman correlation analysis.

### Logistic regression model

In order to establish a diagnostic model for AD classification, logistic regression algorithm was used, and the variance inflation factor (VIF) values for multicollinearity check were all less than 5 and shown in Additional file 1: Table S1. The area under the ROC curve (AUC) was used to evaluate the accuracy of five hub MitoDEGs and logistic regression model. Therefore, we calculated the AUC values of the five hub MitoDEGs to evaluate the accuracy of the diagnostic model using the R package “ROCR” [41]. A nomogram was established to predict the risk of AD based on the feature genes using the “rms” R package [42]. The prediction efficacy of the nomogram was estimated using calibration curves.

### Validation of a diagnostic model

The AUC values of five hub MitoDEGs as diagnostic models were calculated on dataset GSE132903, GSE44770 and GSE33000, respectively, which verified the effectiveness of the diagnostic model.

### Animals

Male WT and APP/PS1 mutant mice aged 10 months old [43] were obtained from the Chinese Academy of Military Sciences (Beijing, China) and had free access to food and water with a comfortable environment. All protocols were approved by the Animal Care and Use Committee of Tianjin Medical University and were performed according to the National Institutes of Health Guide for the Care and Use of Laboratory Animals.

### PC12 neuron culture and handing

PC12 neuron cells were obtained from infrastructure cell line resources in China and cultured in high glucose Dulbecco’s modified Eagle’s medium with 5% fetal bovine serum, and appropriate amounts of penicillin and streptomycin at 37 ℃ and 5% CO_2_. We induced PC12 cells with Aβ1-42 for 12 h, and the final concentration of Aβ1-42 was 7 μM [44].

### Immunofluorescence staining of Aβ1-42 in PC12 cells

PC12 cells were exposed to HiLyte Fluor™488-labeled Aβ1-42 [45] (Table 1) for 12 h. Subsequently, the cells were rinsed twice with PBS and fixed with a 4% paraformaldehyde solution (PFA) for 20 min. Following three washes with PBS, the cells were stained with DAPI (Abcam, UK). Fluorescence images were acquired using a fluorescence microscope (CarlZeiss, Oberkochen, Germany).

### The plasmid transfection

Optic atrophy 1 (OPA1) plasmids and the control empty plasmid vector (GenePharma, Shanghai, China) were transfected into cells using jetPRIME^®^ (Polyplus-transfection S.A, Illkirch, France) according to the manufacturer’s instructions. 2.0 µg DNA plasmid was mixed with 200 µL jetPRIME^®^ buffer, and 4 µL jetPRIME^®^ reagent was added to the above solution, vortex for 1 s, spin down briefly, and incubated for 15 min at room temperature. Then, the transfection mix was added to the 6-well plates with the serum-containing medium and incubated in an incubator for 24-48 h for further experiments.

### RNA extraction and quantitative real-time polymerase chain reaction (qRT-PCR)

Total RNA was extractazed from cultured PC12 cells or the cortex in AD mice using the TransZol Up Plus RNA Kit (TransGEN, Beijing, China) following the manufacturer’s instructions. RNA concentration and quality was measured by Nanodrop Spectrophotometer (Thermo Scientific, Waltham, MA, USA).

Reverse transcription and RT-PCR (mRNA) were performed with corresponding primers (Table 2) using the TransScript^®^ One-Step gDNA Removal and cDNA Synthesis SuperMix (AT311, TransGEN, Beijing, China) and PerfectStart^®^ Green qPCR SuperMix (AQ601, TransGEN, Beijing, China), respectively. The relative value of mRNA transcription was calculated using the 2^−∆∆CT^ formula, and U6/GAPDH was used as the internal control for normalization.

### Detection of mitochondrial reactive oxygen species (mtROS)

To label the mtROS, 1 ml working solution (5 µM) of MitoSOXTM Red (M36008, Invitrogen, Carlsbad, CA, USA) was added into PC12 cells cultured in a 6-well plate and incubated for 10 min at 37 °C in the dark. The cells were washed three times with PBS. To stain the nuclei, a 1 × working solution of Hoechst 33342 (C1027, Beyotime, China) was prepared by mixing 10 μl of the stain with 1 ml of DMEM medium. The working solution was added to the PC12 cells and the cells were incubated in the incubator at 37 °C for 10 min. Then, the cells were washed three times with warm PBS. Images were photographed using a a fluorescence microscope (CarlZeiss, Oberkochen, Germany).

### Detection of mitochondrial membrane potential

Mitochondrial membrane potential (MMP, ∆Ψm) was detected using a JC-1 assay kit (C2003S, Beyotime, China). After the treatment, cultured PC12 cells were washed with PBS. 5 µl JC-1 reagent was mixed with 1 ml JC-1 staining buffer and added to PC12 cells cultured in 6-well plates containing 1 ml medium. The cells were then incubated at 37 ℃ for 20 min. PC12 cells were washed with JC-1 buffer for two times, and fresh medium was added for detection. Images were captured using a fluorescence microscope (CarlZeiss, Oberkochen, Germany) and analyzed using the ImageJ software. When the mt∆Ψ is high in cells, JC-1 aggregates in the mitochondria with a red fluorescence. In cells with low mt∆Ψ, JC-1 is unable to accumulate in mitochondria and remains in the cytoplasm as a monomer, which shows green fluorescence. The value of mt∆Ψ are represented by the ratio of red/green fluorescence intensity.

### Immunofluorescence (IF)

The mice were killed by transcardial perfusion of cold phosphate-buffered saline (PBS) and 4% PFA. Subsequently, the brain tissue was removed completely and fixed with 4% PFA overnight. After gradient dehydration with sucrose, the brain was embedded within the optimal cutting temperature (Sakura, Torrance, CA, USA). The brain samples were cut into slices of appropriate thickness using a − 20 °C frozen slicer for IF staining.

The brain tissue sections were placed at room temperature for 15 min from the – 20 ℃ refrigerator, washed with PBS for 3 times, then treated with 0.3%Triton for 30 min at room temperature, and then incubated with 3% BSA for 60 min. They were then incubated with primary antibodies (Table 1) overnight at 4 ℃. On the second day, the sections were incubated with secondary antibody for one hour after washing with PBS, and then DAPI (Abcam, UK) was used to stain the nucleus. Images were captured using a fluorescence microscope (CarlZeiss, Oberkochen, Germany) and analyzed using the ImageJ software.

### Immunoblotting for protein evaluation

Western blotting for OPA1, caspase3, β-actin was performed, as a previous description [46, 47] (Table 1). The band gray values were measured with ImageJ (National Institutes of health, Bethesda, MD, USA).

### Statistical analysis

R software (version 4.2.2) was used for all bioinformatics statistical analysis and visualization. Values in experiment were expressed as mean ± standard deviation (SD). The t-test was selected to measure the data of the two groups, and one-way ANOVA followed by LSD post hoc test or Tukey’s post hoc test was used for comparisons of multiple groups. Shapiro–Wilk test was used to check the normality of the data. All statistical analyses of experiments were performed by SPSS 26 or GraphPad Prism 9. All experiments were independently repeated at least three times. Significance was defined as P < 0.05.

## Results

### Identification of DEGs in ND and AD samples

**Fig. 1:**
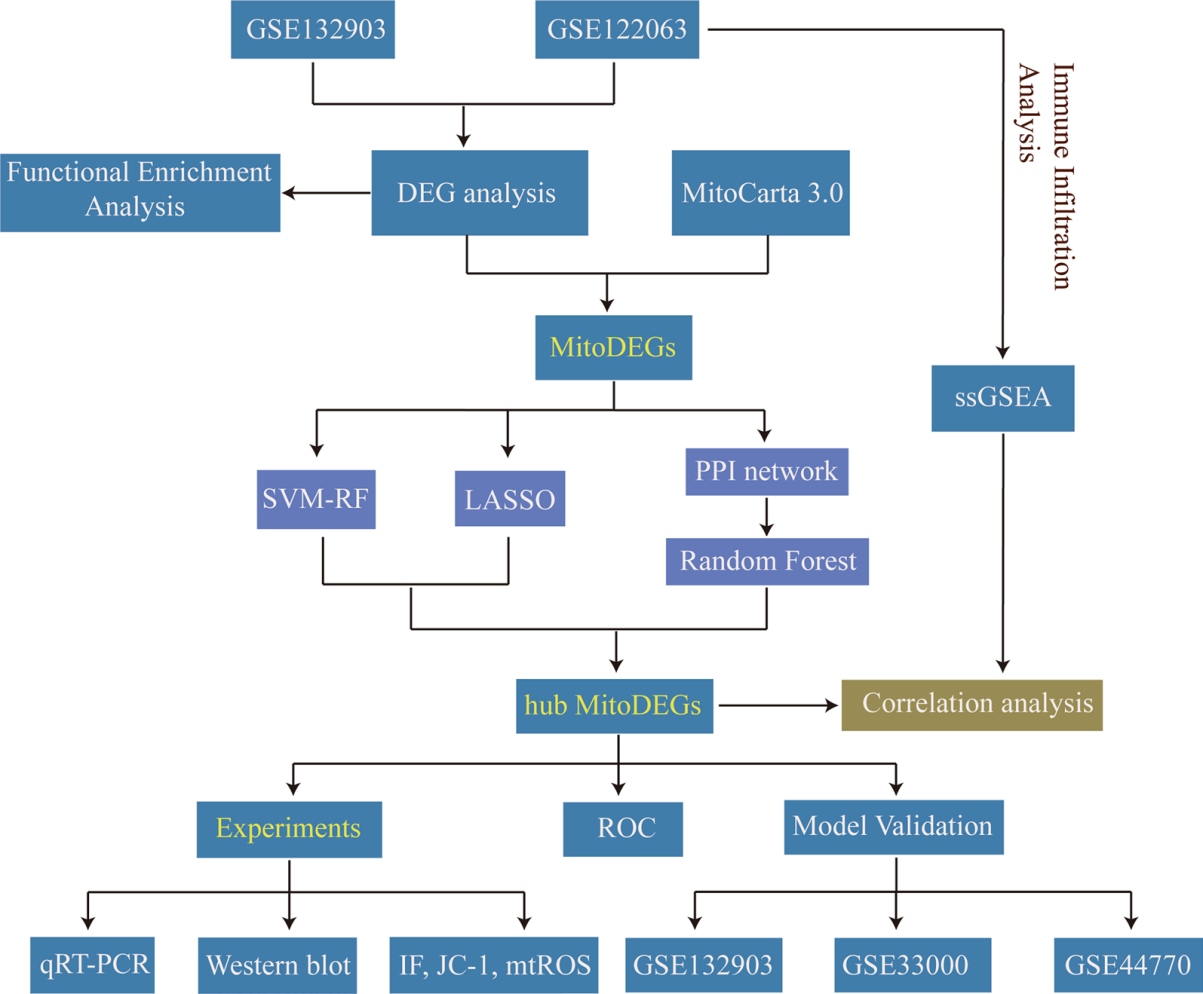
Flow diagram of the study

**Fig. 2:**
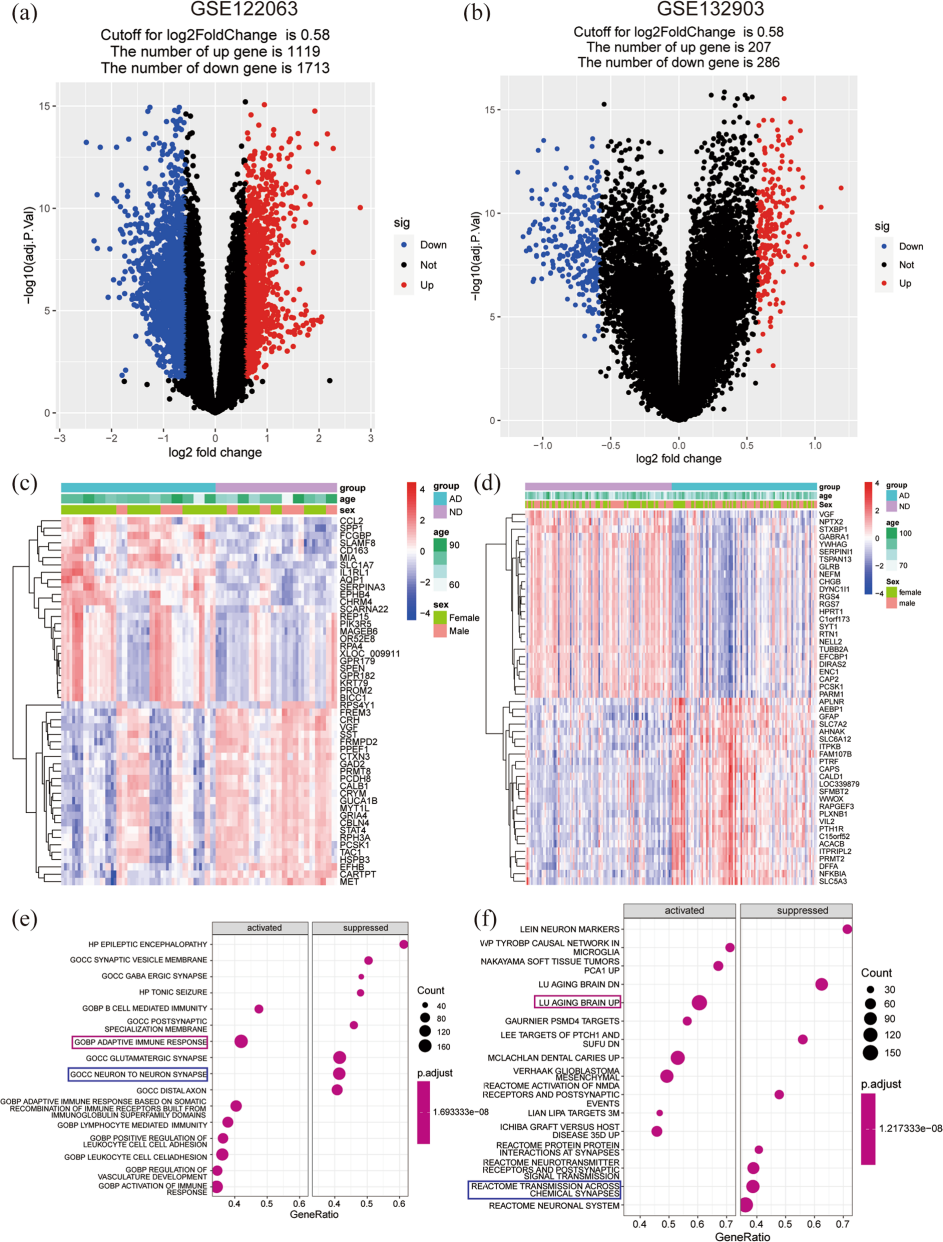
DEGs and enrichment analysis of AD. **a**, **b** Volcano plot of DEGs between AD and ND groups in GSE122063 (**a**) and GSE132903 (**b**). **c**, **d** Heatmap of top 50 DEGs in GSE122063 (**c**) and GSE132903 (**d**). **e**, **f** The top 8 gene sets that areactivated or inhibited in the C5 (**e**) and C2 gene sets of MSigDB (**f**). *AD* Alzheimer’ s disease, *ND* non-demented, Molecular Signatures Database (MSigDB)

The flow diagram of this study is shown in Fig. 1. PCA analysis showed the distribution of the AD and ND samples (Additional file 2: Fig. S1). The details of the selected datasets are shown in Table 3. A total of 18,378 genes were screened after pretreatment in GSE122063, and 2832 DEGs were identified by adj.p.val and Log2FC, including 1119 upregulated genes and 1713 downregulated genes (Fig. 2a). And 493 DEGs were screened in GSE132903, including 207 upregulated genes and 286 downregulated genes (Fig. 2b). The top 25 upregulated and top 25 downregulated genes in the datasets of GSE122063 and GSE132903 are respectively indicated with heatmaps (Fig. 2c, d). The results showed that two groups could be distinguished according to the differential genes.

### Potential functions and pathway of differential expression genes

The top eight significantly activated gene sets and the top eight suppressed gene sets were presented as dotplot from the C5 gene sets of MSigDB. It was found that the immune response was significantly activated, and neuron to neuron synapse was inhibited (Fig. 2e). Similarly, the top eight significantly activated gene sets and the top eight suppressed gene sets in the C2 gene sets of MSigDB were shown as dotplot. Genes related to brain aging were significantly activated, and genes relevant to transmission across chemical synapses were suppressed (Fig. 2f). The details of the top eight gene sets are shown in Additional file 3: File S1.

**Fig. 3:**
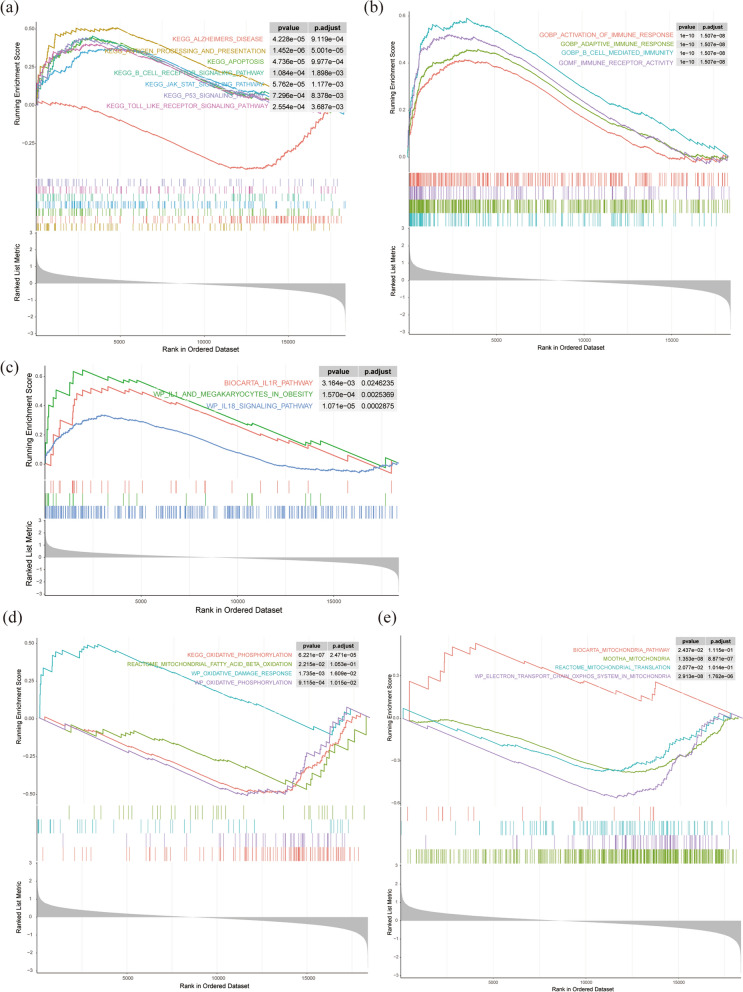
Results of GSEA analysis. **a** The significant GSEA sets in KEGG pathways. **b** The significant GSEA sets in immunity. **c** The significant GSEA sets in Inflammatory pathway. **d** The significant GSEA sets in mitochondrial metabolism. **e** The significant GSEA sets in mitochondria

The GSEA results showed that the antigen processing and presentation, apoptosis, b cell receptor signaling pathway, JAK-STAT signaling pathway, p53 signaling pathway, toll like receptors (TLRs) signaling pathway were enriched in KEGG terms (Fig. 3a). In addition, it also showed that the DEGs were mainly involved in the activation of immune response, IL1R pathway, IL18 signaling pathway, as well as oxidative phosphorylation (OXPHOS), mitochondrial fatty acid β oxidation, oxidative damage response, mitochondria pathway, mitochondrial translation, electron transport chain-oxphos system in mitochondria (Fig. 3b–e). These results suggested that inflammatory responses and mitochondrial metabolism play an important role in AD pathology.

### Analysis of protein–protein interactions network and screening of hub MitoDEGs

**Fig. 4:**
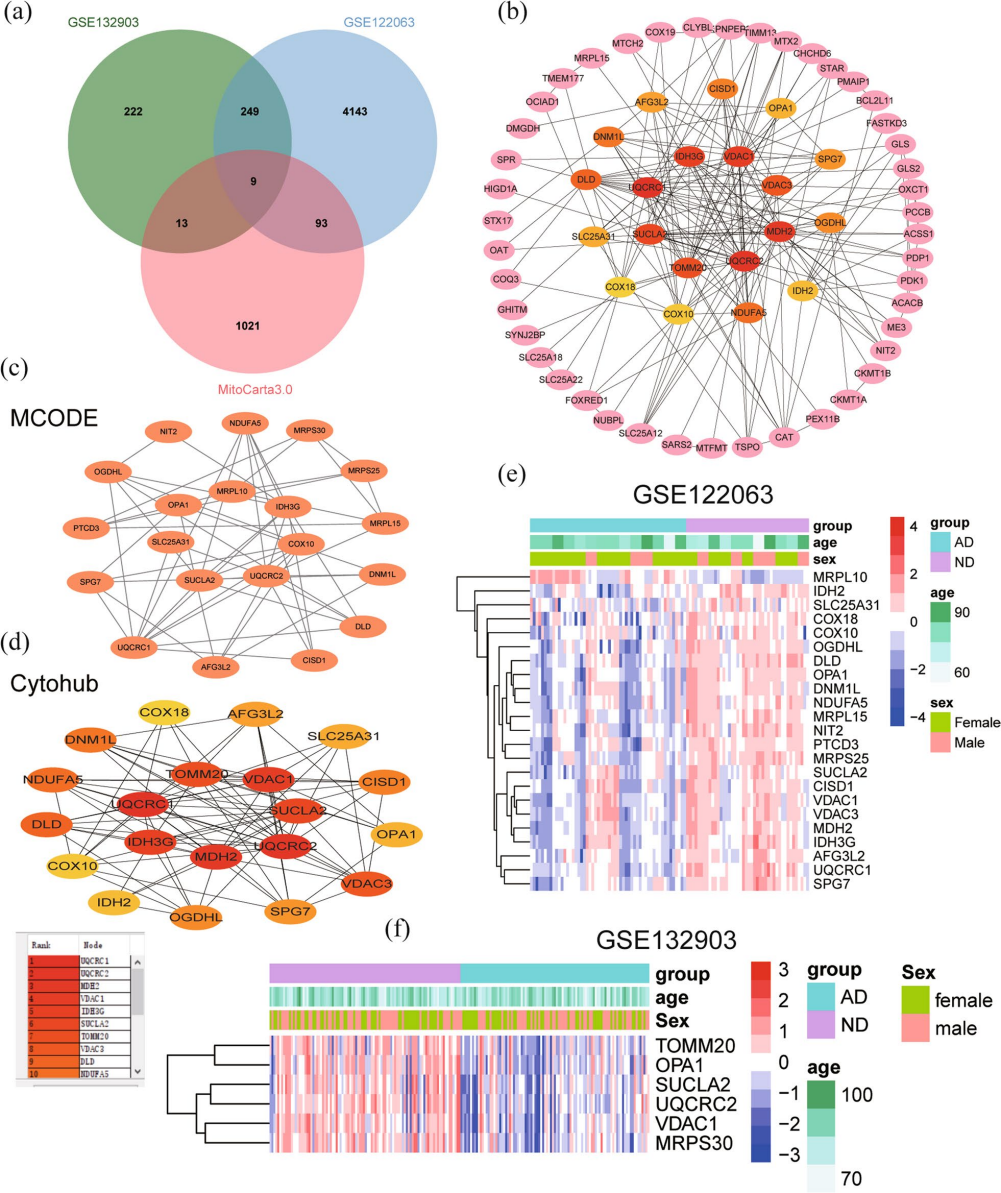
MitoDEGs in AD and PPI network. **a** Venn diagram showed the intersection of DEGs in GSE122063, GSE132903 and mitochondrial genes in MitoCarta 3.0. **b** PPI network of MitoDEGs. **c** A key module with 20 genes as the key gene was screened by MCODE. **d** Top 20 key genes featured by CytoHubba. **e** Heatmap of the expression levels of key MitoDEGs in GSE122063. **f** Heatmap of the expression levels of key MitoDEGs in GSE132903. *PPI* protein–protein interactions

Mitochondria‑related genes were obtained from the MitoCarta3.0 database, and these genes were overlapped with DEGs screened from the GSE122063 and GSE132903 datasets, resulting in 115 overlapping MitoDEGs (Fig. 4a). The expression levels of 115 MitoDEGs were shown in Additional file 4: File S2. PPI analysis of 115 MitoDEGs using STRING database and network visualization with Cytoscape (Fig. 4b). Important modules were identified with Cytoscape's plugin MCODE and a module consisting of 20 nodes and 53 edges was selected (Fig. 4c). Meanwhile, top 20 hub genes were identified from the PPI network using the plugin CytoHubba (Fig. 4d). A total of 26 genes were obtained after the combination, including 23 genes in GSE122063 and 6 genes in GSE132903 (Fig. 4e, f).

### Screening of key genes and correlation analysis with immune cells

**Fig. 5:**
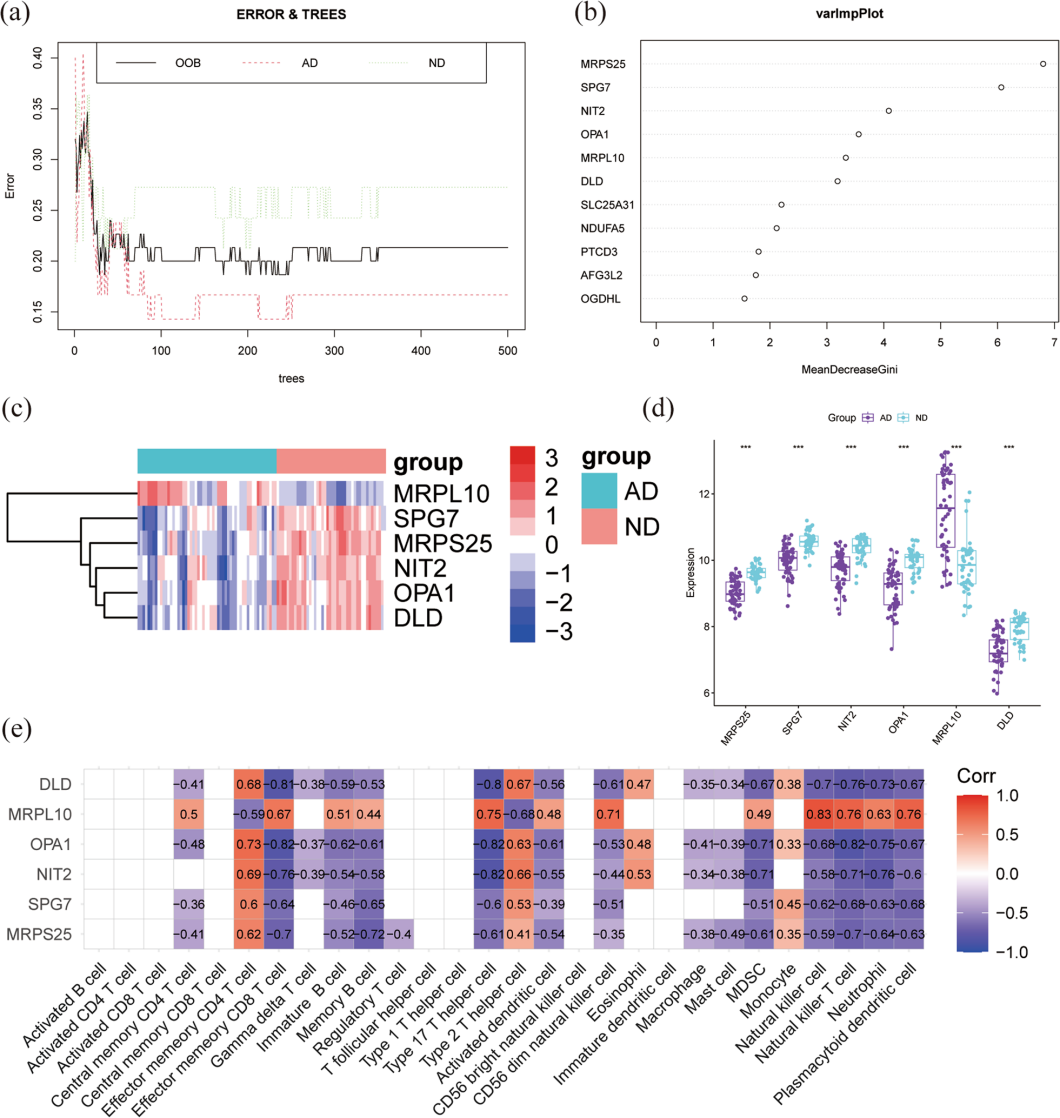
Screening of key genes by random forest and correlation analysis with immune cells. **a** The correlation plot between the number of RF trees and model error. **b** Results obtained by Gini coefficient method in random forest classifier, and significant values greater than 3 were selected as key MitoDEGs. **c**, **d** The expression levels of the six key MitoDEGs of AD samples in GSE122063. **e** The correlation between six key MitoDEGs and immune cells. RF, random forest

In order to screen the key genes, we fed the 23 DEGs obtained from the PPI analysis into the RF classifier. The top six genes with significant values greater than 3 were identified as candidates for further analysis (Fig. 5a, b). MRPL10 was significantly highly expressed in the AD group, while SPG7, MRPS25, NIT2, OPA1 and DLD were poorly expressed in the AD group (Fig. 5c, d), as shown in the heatmap. In the six MitoDEGs, SPG7, MRPS25, NIT2, OPA1 and DLD were found to be negatively correlated with memory B cell, effector memory CD8 T cell, activated dendritic cell, natural killer T cell, type 17 T helper cell, MDSC, Neutrophil, plasmacytoid dendritic cell. MRPL10 was found to be positively correlated with these immune cells (Fig. 5e).

**Fig. 6:**
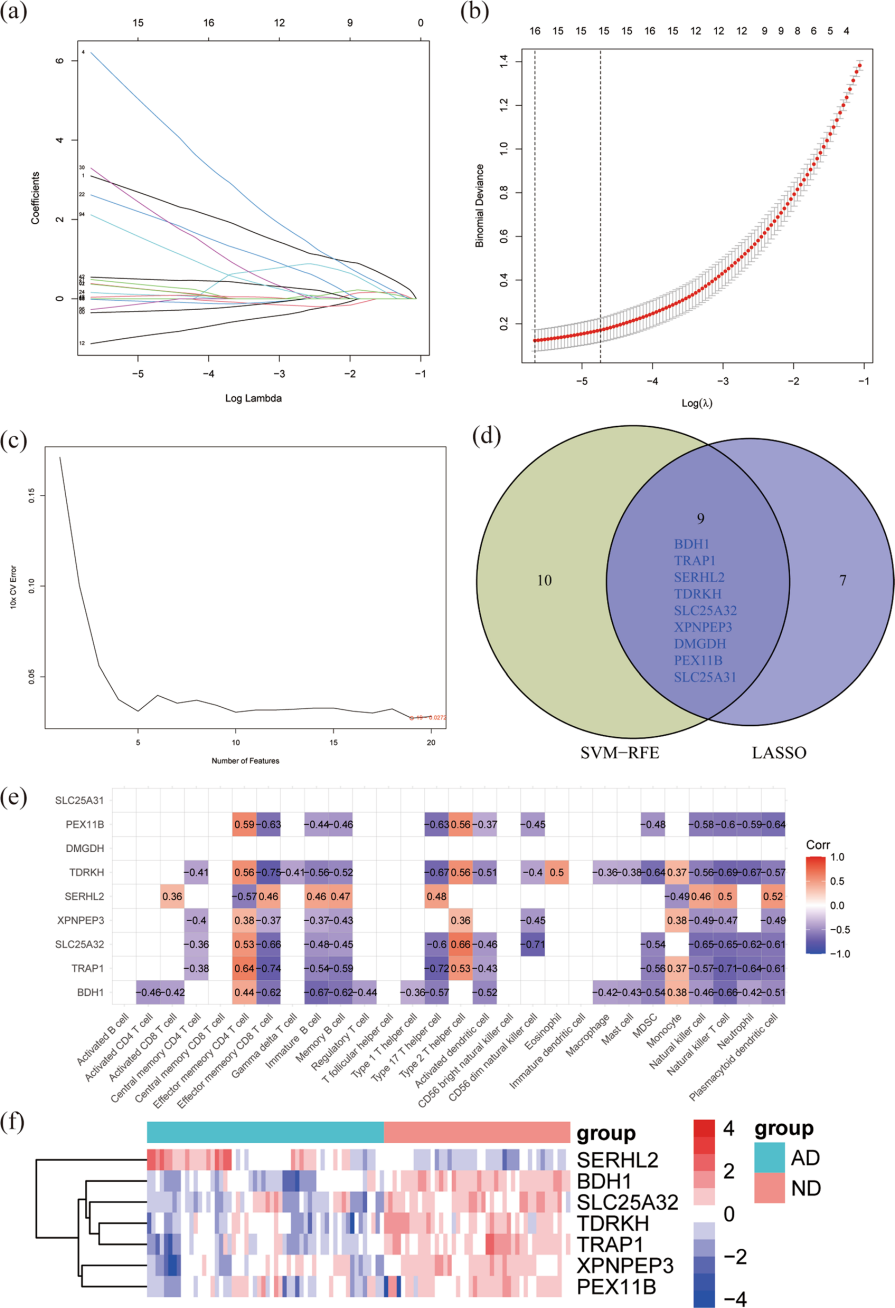
Identification of feature MitoDEGs using machine learning algorithms and correlation analysis with immune cells. **a** Selection of the optimal parameter (lambda) in the LASSO model. **b** Coefficient distributions of the 16 most correlated MitoDEGs determined by the optimal lambda. **c** SVM-RFE algorithm screened the most relevant MitoDEGs. **d** Venn diagram demonstrated the intersection of feature MitoDEGs obtained by the two Machine learning algorithms. **e** The correlation between nine key MitoDEGs and immune cells. **f** Heatmap of the expression levels of seven key MitoDEGs in AD. LASSO, least absolute shrinkage and selection operator. SVM-RFE, Support vector machine recursive feature elimination

To further improve the quality of key MitoDEGs of AD, we used two different machine learning algorithms to screen genes. We found 16 feature MitoDEGs using the LASSO method (Fig. 6a, b), and 19 feature genes were obtained using the SVM-RFE algorithm (Fig. 6c). The intersection of genes obtained using two machine learning algorithms was used to identify nine mitoDEGs for subsequent studies (Fig. 6d). In order to further explore the potential relationship between nine MitoDEGs and immune cells. SLC25A31 and DMGDH were found to have no associations with immune cell subsets by Spearman method (Fig. 6e). Therefore, these two genes were excluded for the further research. The expression levels of the seven MitoDEGs (BDH1, TRAP1, SERHL2, TDRKH, SLC25A32, XPNPEP3, PEX11B) in AD and ND samples were shown (Fig. 6f). These results suggested that these seven genes were associated with memory B cell, effector memory CD8 T cell, activated dendritic cell, natural killer T cell, type 17 T helper cell, MDSC, Neutrophil, plasmacytoid dendritic cell.

### Immune cell infiltration in AD

**Fig. 7:**
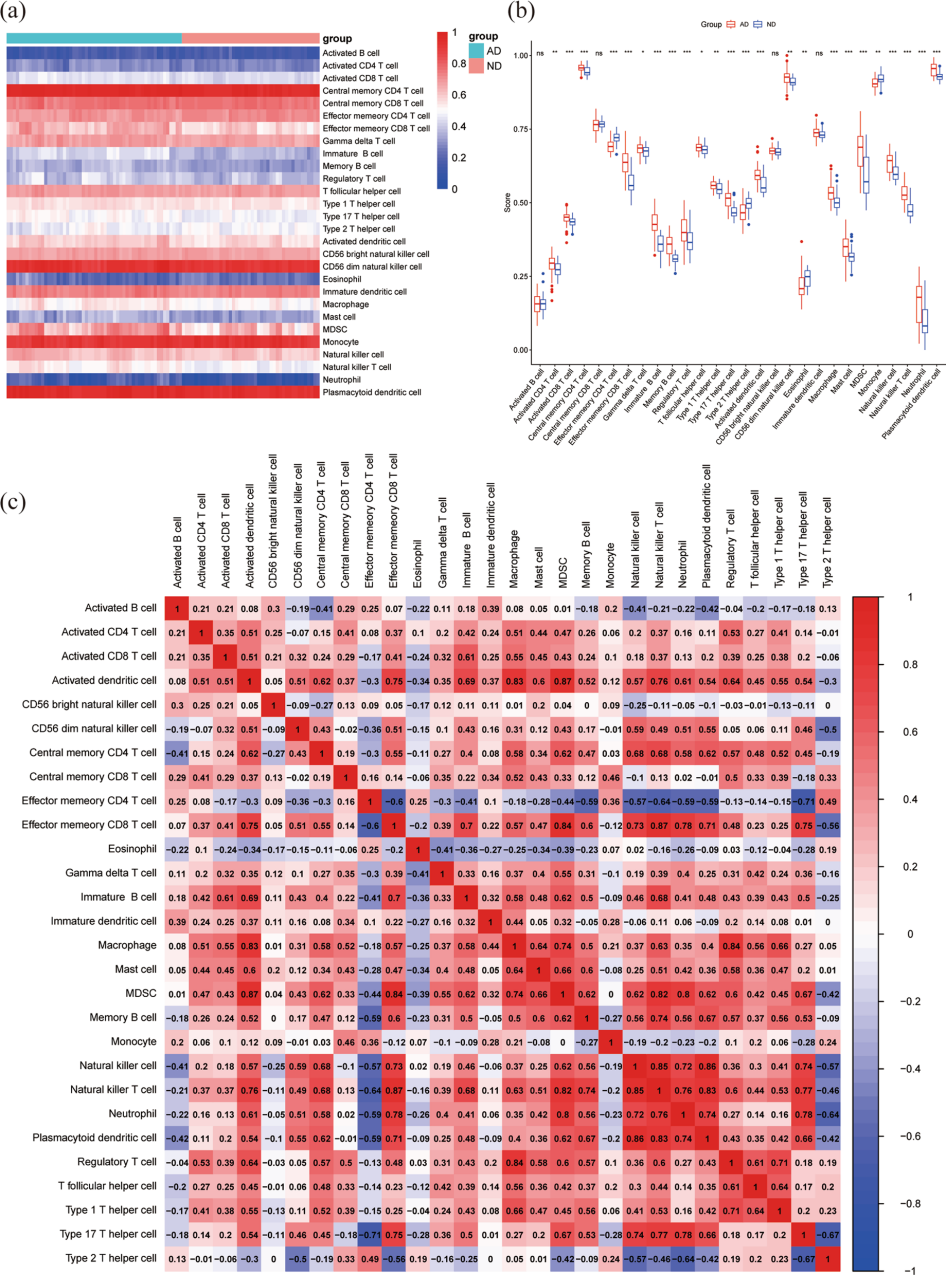
The infiltration of immune cell types of the AD and ND. **a** Heatmap of the proportions of 28 immune cells in the AD and ND groups. **b** The boxplot of the immune cell proportions in AD and ND. **c** The correlation matrix of immune cell proportions

Twenty eight immune cell infiltrates were analyzed in the AD and ND groups, and 24 kinds of immune cell subsets were found to be different between the AD and ND groups (Fig. 7a, b). Among them, the proportion of macrophage, activated CD8 T cell, activated CD4 T cell, effector memory CD8 T cell, memory B cell, natural killer cell, natural killer T cell, type 17 T helper cell, Neutrophil, MDSC, plasmacytoid dendritic cell, regulatory T cell, activated dendritic cell increased in AD. And the proportion of effector memory CD4 T cell, type 2 T helper cell, monocyte, eosinophil decreased. Additionally, further analysis of the infiltration of immune cells demonstrated complex correlations between cells (Fig. 7c), such as effector memory CD8 T cell/natural killer T cell (0.87), activated dendritic cell/MDSC (0.87), natural killer cell/natural killer T cell (0.85), plasmacytoid dendritic cell/natural killer cell (0.86), macrophage/regulatory T cell (0.84), activated dendritic cell/macrophage (0.83). The positive/negative associations between mitoDEGs/hub mitoDEGs and immune cells were added and shown in Additional files 5: Fig. S2.

### Validation of a diagnostic model for hub MitoDEGs

**Fig. 8:**
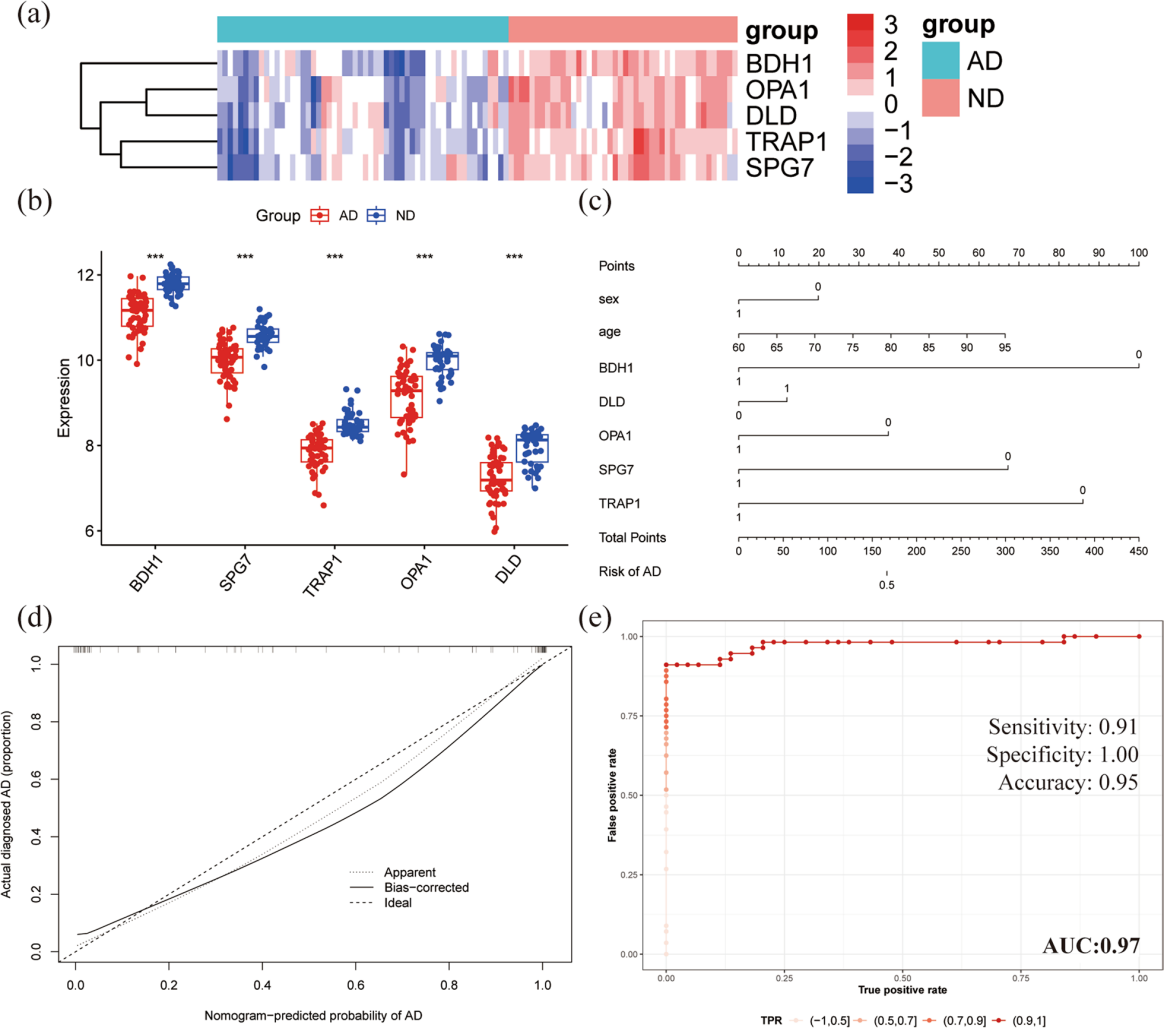
Nomogram model of AD patients was constructed based on clinical characteristics. **a**, **b** The expression levels of the five hub MitoDEGs in AD and ND samples. **c** A nomogram model combined with based on sex, age and five hub MitoDEGs was constructed to predict the risk of AD patients. **d** The calibration curve of the nomogram tests the predictive performance of the model. **e** ROC curves analysis of GSE122063 for the diagnostic model including the five hub MitoDEGs

Five hub MitoDEGs (BDH1, TRAP1, OPA1, DLD and SPG7) were finally screened by finding the biological functions of the above key genes. The expression levels of the five MitoDEGs in AD and ND samples were shown (Fig. 8a, b). The expression levels of hub mitoDEGs were also verified in GSE132903, GSE44770 and GSE33000, as shown in in Additional files 6: Fig. S3. The nomogram plots were established to predict the risk of AD progression by combining these five hub genes and clinical feature (sex, age). The details of the hub MitoDEGs and clinical feature are shown in Additional file 7: File S3. Each gene and the clinical feature correspond to a scoring criterion (Fig. 8c). The calibration curve of the nomogram confirmed the good predictive performance of our model (Fig. 8d). In addition, the ROC curve analysis was performed to evaluate the predictive power of nomogram. The overall AUC of this model is 0.974, indicating that hub MitoDEGs had a high diagnostic value (Fig. 8e).

**Fig. 9:**
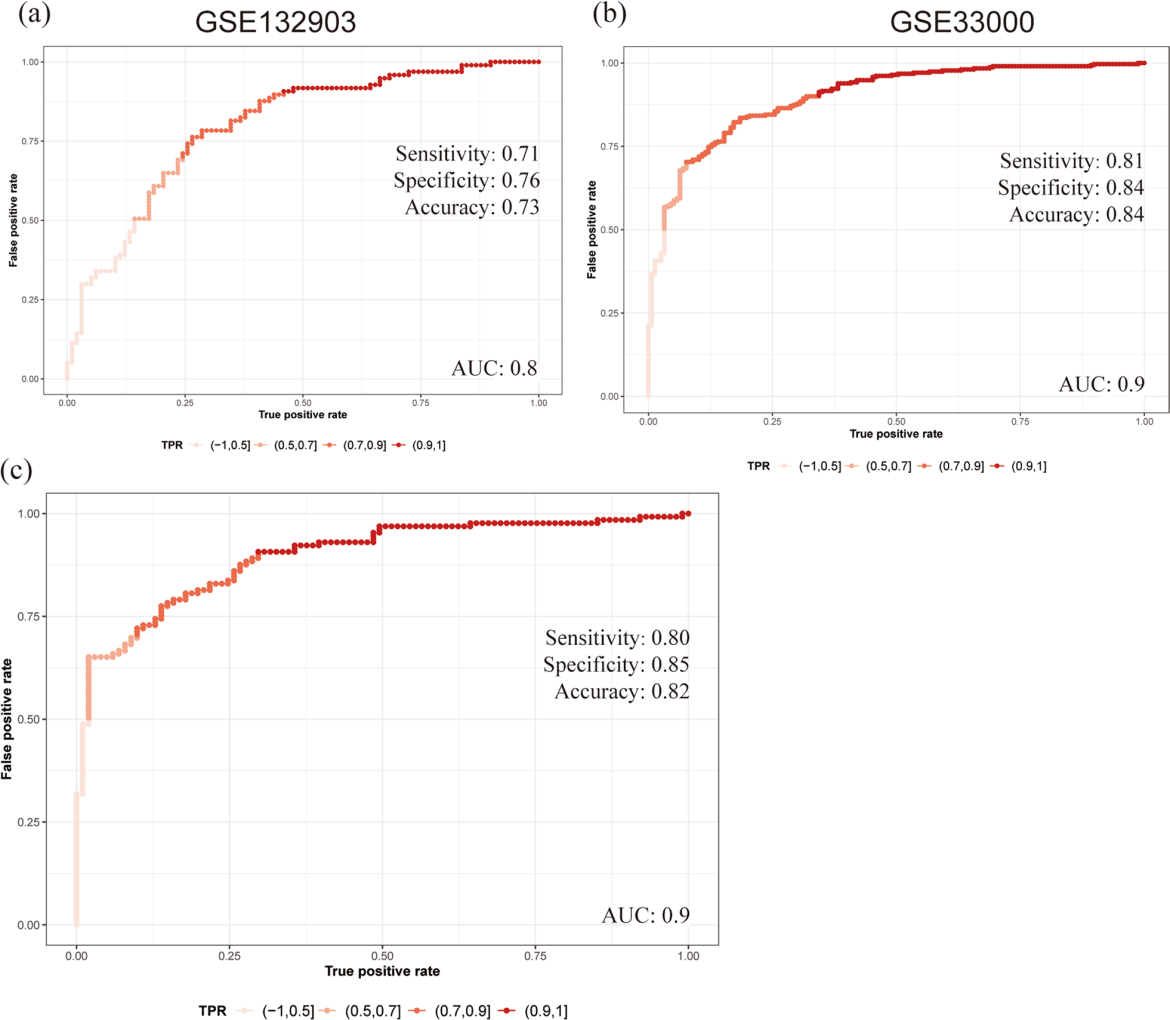
ROC curves were used to evaluate the diagnostic efficacy of the five hub MitoDEGs. **a** GSE132903 datasets. **b** GSE33000 dataset. **c** GSE44770 dataset

Finally, three independent datasets were used to verify the accuracy of the model. AUC values for hub MitoDEGs were calculated using the logistic regression algorithm. The AUC of 0.796 in GSE132903 dataset, the AUC of 0.898 in GSE33000 dataset and the AUC of 0.897 in GSE44770 dataset, indicating that the model is stable for the diagnosis of AD (Fig. 9a–c). In summary, we developed an AD diagnostic model using the five hub MitoDEGs.

### Expression of hub MitoDEGs in PC12 cells and the brain tissue of AD mice

**Fig. 10:**
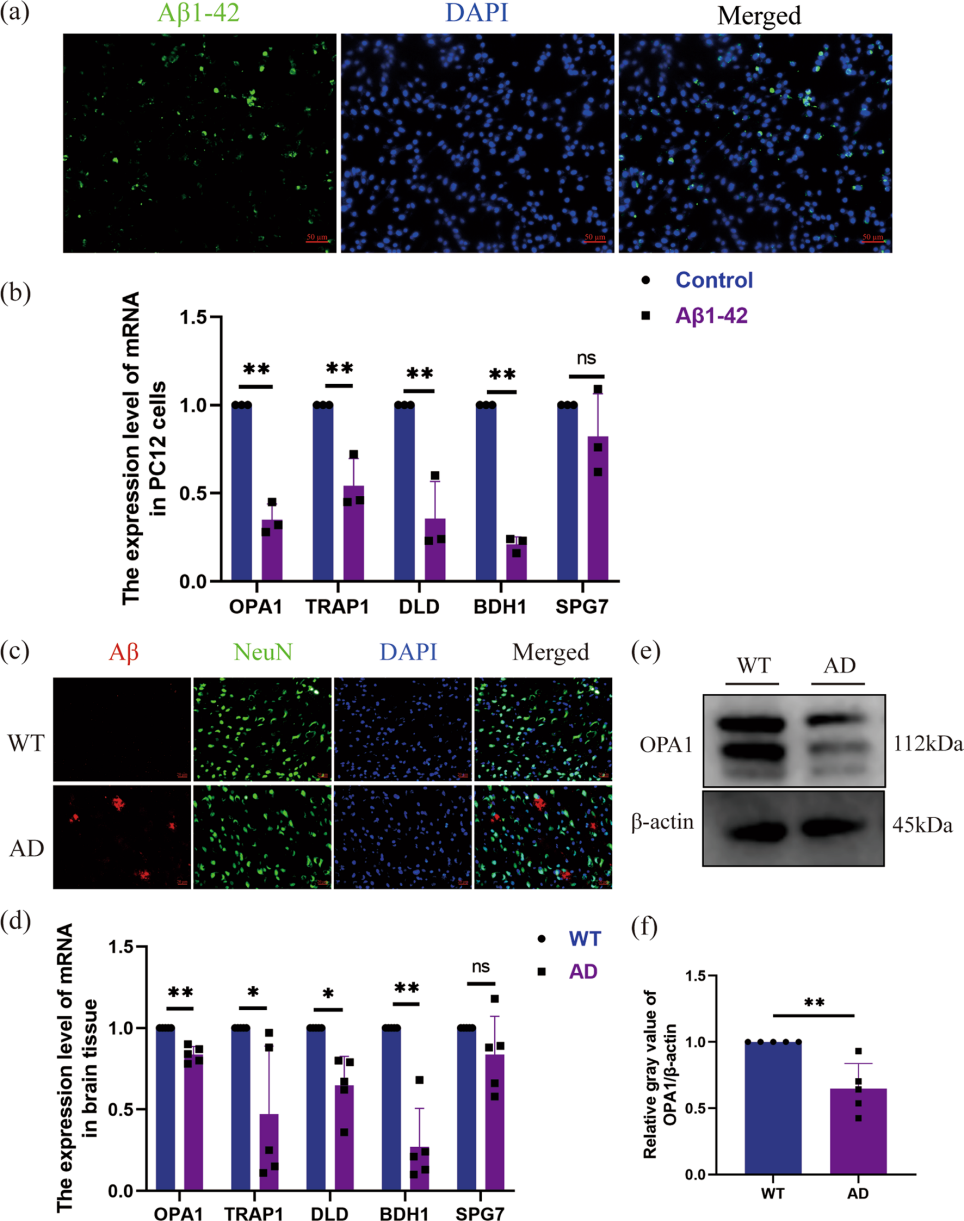
Validation of mRNA expression levels of five hub MitoDEGs in neurons and AD mice. **a** Immunofluorescence staining of PC12 cells treated with Aβ1-42 for 12 h, showing the presence of Aβ1-42 in the neurons. **b** Relative mRNA expression of the five hub MitoDEGs was analyzed in PC12 cells treated with Aβ1-42 for 12 h by qRT-PCR. **c** Immunofluorescence staining of Aβ expression in the AD mouse models, indicating the deposition of Aβ plaques. **d** The mRNA expression levels of BDH1, TRAP1, OPA1, DLD and SPG7 was detected in the cortex of AD mice. **e**, **f** Representative western blot (**e**) and quantitative data (**f**) of OPA1 protein in AD mice. *p < 0.05; **p < 0.01; ***p < 0.005. qRT-PCR, quantitative real-time reverse transcription-polymerase chain reaction. *Aβ* β-amyloid

To determine whether Aβ1-42 entered the neurons, immunofluorescence staining of PC12 cells treated with Aβ1-42 for 12 h showed the presence of Aβ1-42 in the neurons (Fig. 10a). The expression of five hub MitoDEGs in PC12 neurons was detected by qRT-PCR. The mRNA expression of BDH1, TRAP1, OPA1, DLD was significantly decreased in the Aβ1-42 group compared with the control group, while the expression of SPG7 was not significantly different between the two groups (Fig. 10b). Similarly, immunofluorescence staining of Aβ protein expression in AD mouse cortex indicated that Aβ protein was highly expressed in AD mice (Fig. 10c). The mRNA levels of BDH1, TRAP1, OPA1, DLD also decreased significantly in the cortex of AD mice, and the expression level of SPG7 showed a downward trend (Fig. 10d). The OPA1 protein levels also decreased significantly in the cortex of AD mice (Fig. 10e, f). These results indicated that the expression levels of five hub MitoDEGs were consistent with the bioinformatics results.

**Table 1:** List of the primary antibodies used in this study

Antibody	Catalogue number	Brand	Application	Dilution	Species	MW (kDa)
OPA1	27733-1-AP	Proteintech	WB	1:1000	Rabbit	112
Caspase3	9662	CST	WB	1:1000	Rabbit	17, 19, 35
β-actin	4970	CST	WB	1:1000	Rabbit	45
β-Amyloid	ab201060	Abcom	IF	1:100	Rabbit	NA
NeuN	ab104224	Abcom	IF	1:1000	Mouse	NA
β-Amyloid (1-42)	AS-60479-01	AnaSpec	IF	1 µg/ml	NA	NA

*Abcam* Abcam, Cambridge, MA, USA, *CST* Cell Signaling Technology, Danvers, MA, USA, *MW* molecular weight, *NA* not applicable, *WB* western blot, *IF* immunofluorescence

**Table 2:** Primer sequences of mRNA for qRT-PCR

	Primer sequence, 5'–3'	
	Forward	Reverse
Gene (mouse)		
BDH1	CCTCTGTCATCAACGCTGTCAC	ATCCGAAGCCACCAGTAGTAGTC
TRAP1	GATAGCAGCAGGACTCGTTGATG	CAGTGTTTCTCCAGGACCTTGAC
OPAl	AGGATGGTGCTCGTGGACTTG	CACAGGATGATGGCGTTAGGATTC
SPG7	AACACGGAAGAGGAGCAGACC	ATCAGAGCATTGTCCAGAACATCAG
DLD	TGCCATCAAATCTGCCCAGTTAG	ACGTTCAAGCATGTTCCACCTAG
GAPDH	GCCAAGGCTGTGGGCAAGGT	TCTCCAGGCGGCACGCAGA
Gene (rat)		
OPAl	AGGATGGTGCTGGTGGACTTG	CACAGGATGATGGCGTTAGGATTC
BDH1	ACCTACACCAGTCAGGCAGATG	CCAAAGAGAACCCAAATCCAGAGTC
TRAP1	GCATTGTGACCACCGCTGAG	TCGGCTGGCGTAGTCTGATAAG
SPG7	GAAGAAGAGAGGAGGCGGAAGG	TCACGATGGCGATGATGAACAAG
DLD	GATGGCAGCACTCAGGTTATTGG	AAGCTCCCGTAGACGACACTATAG

**Table 3:** The details of the selected datasets

Location	Datasets	Platform	Type	The number of ND group	The number of AD group	Female	Male	Mean age of ND (year)	Mean age of AD (year)
Frontal and temporal cortex	GSE122063	GPL16699	Microarray	44	56	68	32	78.82	80.88
Middle temporal gyrus	GSE132903	GPL10558	Microarray	98	97	96	99	84.98	85.02
Prefrontal cortex, visual cortex and cerebellum	GSE44770	GPL4372	Microarray	101	129	86	144	62.12	80.09
Prefrontal cortex	GSE33000	GPL4372	Microarray	157	310	209	258	63.52	80.6

### OPA1 overexpression alleviates damage of mitochondria and neuronal apoptosis caused by Aβ1-42

**Fig. 11:**
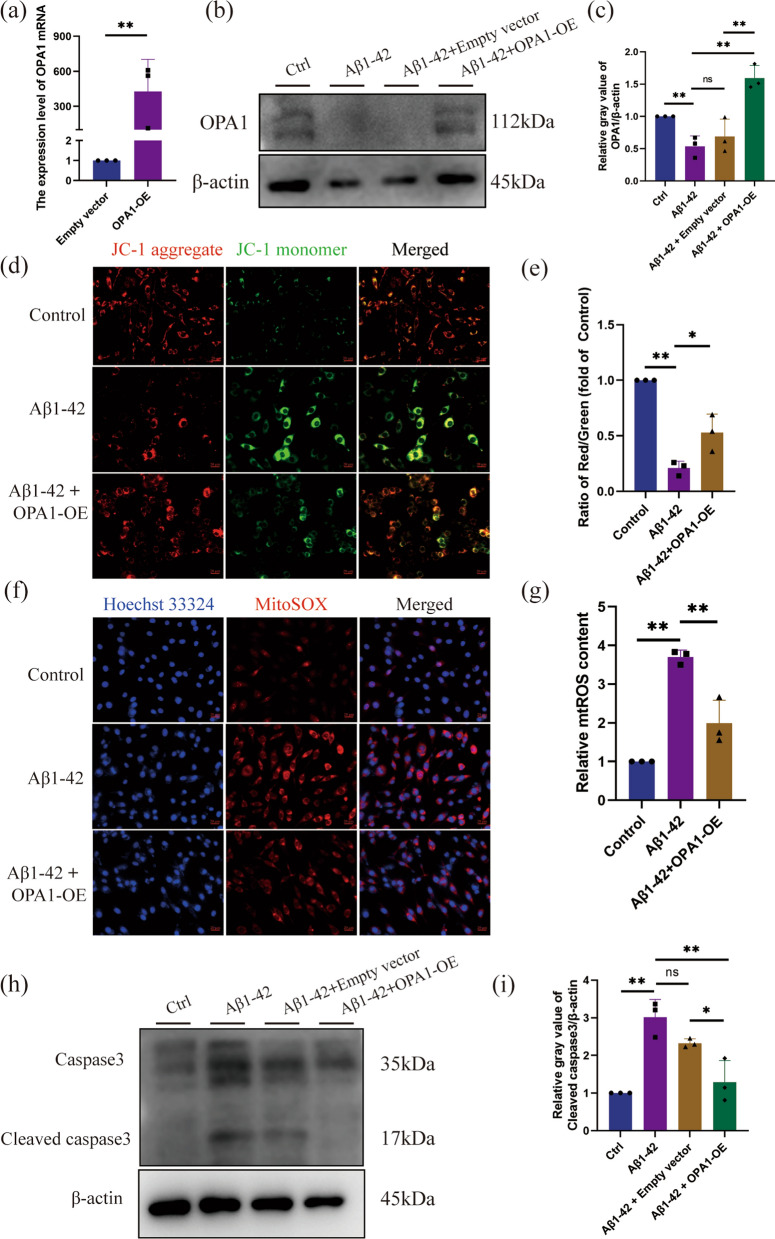
Overexpression of OPA1 attenuated Aβ1-42 induced mitochondrial dysfunction and neuronal apoptosis in PC12 cells. PC12 cells were transfected with OPA1 overexpression plasmids or empty vector for 48 h followed by treating with Aβ1-42 (7 μM/ml) for 12 h. **a** Quantification of OPA1 mRNA expression. **b**, **c** Representative western blot (**b**) and quantitative data (**c**) of OPA1 protein in PC12 cells. **d** Representative images of JC-1 staining, showing the red fluorescence of JC-1 aggregate and green signal of monomer. Scale bar: 20 µm. **e** Quantification of the ratio of JC-1 aggregate to JC-1 monomer. **f** Representative images of MitoSOX staining for mitochondrial ROS. Scale bar: 20 µm. **g** Quantification of MitoSOX fluorescence intensity. **h**, **i** Representative western blot (**h**) and quantitative data (**i**) of Cleaved caspase3 protein in PC12 cells. Data are depicted as the mean ± SD from three independent experiments. *p < 0.05, **p < 0.01, ***p < 0.001. *Ctrl* control, *Empty vector* control empty plasmid vector, *OPA1-OE* OPA1 overexpression plasmids

OPA1 has been identified as a promising gene for research in adipose tissue [48], ischemic stroke [49], prion diseases [50] and so on, and its overexpression has been shown to improve mitochondrial dysfunction [50]. Therefore, we chose to investigate OPA1 for validation. We demonstrated that OPA1 protein was significantly decreased in PC12 cells treated with Aβ1-42. Plasmids with overexpression of OPA1 were transfected into neurons, and the transfection efficiency was obvious (Fig. 11a–c). OPA1 overexpression alleviated the Aβ1-42 induced loss of ∆Ψm (Fig. 11d, e). Consistently, OPA1 overexpression inhibited Aβ1-42 induced mitochondrial ROS generation in PC12 cells (Fig. 11f, g). The cleaved caspase 3 levels increased significantly in Aβ1-42-induced PC12 cells, while cleaved caspase 3 levels decreased significantly after OPA1 overexpression (Fig. 11h, i). Collectively, these results revealed the critical role of OPA1 in maintaining mitochondrial stability and neuronal apoptosis.

## Discussion

There are two forms of Alzheimer's disease: early-onset (familial) and late-onset (sporadic). Familial AD typically occurs between the ages of 30 and 50, and accounts for 1–2% of all AD cases. Familial AD is caused by mutations in the Aβ precursor protein, presenilin-2and presenilin-1 genes, resulting in overproduction of Aβ plaques [51, 52]. Late-onset AD involves a variety of factors including lifestyle, traumatic brain injury, obesity, hypertension, diabetes, depression, and epigenetic factors [53]. Similarly, APOE4 genotype and age are important risk factors for AD [1, 54]. It has been pointed out that the presence of the toxic proteins is thought to activate microglia to remove excess proteins and dead cells [55]. When microglia cannot maintain their balance, chronic inflammation occurs in the brain. Therefore, inflammation is largely a biomarker of AD.

Given the close relationship between the immune microenvironment status and the occurrence and progression of AD [5, 10], as well as the alterations of mitochondrial metabolism and mitoophagy processes in immune cells [17, 18, 22, 56], it is crucial to identify potential targets of mitochondrial genes to guide the treatment of AD. In our study, we used bioinformatics methods to comprehensively investigate the DEGs of AD and ND from the GEO database and found that the immune response and brain aging were enriched in AD patients. Moreover, neuron to neuron synapse and the transmission across chemical synapses were inhibited in AD patients. Further analysis revealed that IL-1β, IL-18 inflammatory pathways, oxidative damage were activated, and mitochondrial OXPHOS and fatty acid oxidation were decreased in AD samples.

Peripheral immune disorders and peripheral-central immune crosstalk have been investigated for their important roles in the pathogenesis and progression of AD [8]. Several researchers have demonstrated cytotoxic effects of CD8^+^ efferent memory CD45RA^+^ (T_EMRA_) cells, Th1 and Th17 in CD4 T cells in AD pathology, which is consistent with our study [57, 58]. Natural killer (NK) T cells have the ability to rapidly induce cell apoptosis through cytotoxic granules and release of inflammatory factors such as TNF-α, INF-γ. When stimulated, NK cells can activate other immune cells, triggering an immune cascade reaction. Furthermore, alterations in NK cell subsets are closely associated with the progression of Alzheimer's disease (AD) [11, 59]. Similarly, inhibition of neutrophils also reduces systemic chronic inflammation and cognitive decline at various stages of AD progression [9, 60].

In this study, we found that compared with the ND group, the AD group had significant changes in the proportion of immune cell infiltration, including macrophage, regulatory T cell, activated CD8 T cell, memory B cell, activated dendritic cell, activated CD4 T cell, natural killer T cell, type 17 T helper cell, Neutrophil, MDSC. CD4 T cells play an important role in neurodegenerative diseases, such as Parkinson's disease (PD) [61], AD [62], stroke [63], and Lewy body dementia [64]. CD4 T cells mainly influence the function of mature microglia to neuronal synapses [65]. Recently, researchers have found that there are many more T cells, especially cytotoxic T cells, in the mice of tauopathy than in the mice with amyloid deposition or control mice. T cells are most abundant in areas with the most severe tau pathology and the highest concentration of microglia. Activated microglia release molecular compounds that activate and draw T cells from the blood into the brain, and T cells release compounds that push the microglia toward a more pro-inflammatory phenotype. Together, these two types of immune cells create an inflammatory environment that is primed for neuronal damage [66]. In AD, Aβ deposition and p-tau accumulation induce parenchymal innate immune activation, affecting the integrity of the blood–brain barrier (BBB), CSF/ISF flow and lymphatic drainage, further leading to the antigen-presenting microglia, expansion of IFN-reactive, the increase of inflammatory cytokines and antigen accumulation, as well as parenchymal T-cell infiltration, T cell receptor (TCR) clonal expansion in the brain parenchyma and border areas [4]. In the innate immune response, TLRs recognize molecular patterns associated with microbial pathogens and damage, promoting NF-kB signaling and the activation of inflammation [4, 67]. The increase in TLRs signaling pathway in AD samples was also found in our study.

Subsequently, we discovered the strongly relationship between hub mitoDEGs and immune cells, as well as immune microenvironment and AD pathology, and finally identified five hub MitoDEGs (BDH1, TRAP1, OPA1, DLD, SPG7) through in-depth analysis of three machine learning algorithms, which was of great significance for the search for pathological biomarkers and promising therapeutic intervention targets for AD. Although there have been some studies on the immune microenvironment related to AD [10], and the gene sets (CXCR4, PPP3R1, HSP90AB1, CXCL10, and S100A12) that are responsible for immune filtration have been indicated. Inflammatory gene markers activated in immune cells could induce harmful neuroinflammatory programs and contributed to neurodegenerative environments [68]. However, we investigated mitochondrial genes associated with the immune microenvironment. These hub MitoDEGs (OPA1) regulated mitochondrial ROS, MMP, and neuronal apoptosis. In the same way, epigenetic silencing may also promote neuron survival by eliminating the potential mediator of neuron death [69]. In short, under different physiological and pathological conditions, unique patterns of altered gene expression may reduce or promote disease progression by affecting immune cells, epigenetic inheritance, or simultaneously activating multiple synergistic regulatory mechanisms.

One of the important pathogeneses of AD is the disorder of mitochondrial metabolism, which can control synaptic transmission and information exchange. Mitochondrial metabolism has an enormous impact on the fate and function of immune cells. Microglial lipid metabolism is involved in microglial activation, phenotypic transitions and functions, such as phagocytosis, inflammatory signaling, and migration [70]. In addition, the interaction of Aβ and P-tau with dynamin-related protein 1 (Drp1) is the key factors in mitochondrial fragmentation, damage of mitochondria and synapsis, eventually possibly resulting in neuronal damage and cognitive deficit [71, 72]. OPA1 is an important intima fusion protein regulated by two membrane proteases, OMA1 and YME1L1. Studies have pointed out that inhibition of OPA1 enhances the progression of neurodegenerative diseases, and OPA-1 regulates apoptosis resistance of OXPHOS IL-17-producing CD4 T cells by regulating mitochondrial fusion and limiting mitophagy [73]. In conclusion, the OPA1 may part acts on immune cells to maintain mitochondrial homeostasis in patients with AD. Overexpression of OPA1 is associated with adipocyte browning, which is beneficial to improve glucose tolerance and insulin sensitivity [48]. However, in the AD model, OPA1 may not affect cognitive function by altering tau's phosphorylation [74]. Our study demonstrated that up-regulation of OPA1 restored mitochondrial membrane potential and reduced neuronal apoptosis, which was consistent with previous studies [50], suggesting that OPA1 may alter AD disease progression by affecting neuronal apoptosis rather than tau's phosphorylation.β-hydroxybutyrate dehydrogenase (BDH1), as a main rate-limiting enzyme of ketone metabolism, controls the transformation between acetoacetic acid (AcAc) and β-hydroxybutyrate (BHB). BHB acts as an alternative carbon source for maintaining the redox balance, including the production of amino acids, OXPHOS, and glutathione. The ability of BHB to enhance CD4 + T cell metabolism and promote T cell responses depends on BDH1 [75]. In addition, it was found that BDH1 is significantly down-regulated in glioblastoma [76] and has an important role in metabolic regulation in the liver [77]. Tumor necrosis factor receptor-associated protein 1 (TRAP1), a member of the chaperone family of heat shock protein 90 (HSP90), is predominantly present in mitochondria. TRAP1 is a regulator factor of oxidative stress-induced cell death, redox homeostasis and unfolded protein response [78]. Giffard et al. demonstrated that TRAP1 overexpression reduced ROS production, maintained mitochondrial membrane potential and increased preservation of ATP levels in oxygen-glucose deprived neurons and astrocytes [79, 80]. Moreover, TRAP1 may be a causative gene in PD, which needs to be further confirmed [81, 82]. Therefore, it is very meaningful to find this target in AD and explore it in depth. Spastic paraplegia type 7 (SPG7) mutations are a common cause of hereditary spastic paraplegia, resulting in mitochondrial dysfunction, including decreased mitochondrial membrane potential, reduced OXPHOS, decreased ATP, and increased mitochondrial stress [83]. The lack of paraplegin, a protein encoded by SPG7, impairs the opening of mitochondrial permeability transition pore (mPTP) by increasing the expression and activity of sirtuin3, thereby increasing the concentration of Ca^2+^ and reactive oxygen species in the matrix and destroying mitochondrial homeostasis, leading to the disorders of synaptic transmission disorders [84]. In the present study, we found a downward trend in SPG7, but there was no statistical difference, which may be caused by different species of samples or the heterogeneity of samples. Dihydrolipoamide dehydrogenase (DLD), as a cuproptosis-related gene [85], is associated with steatosis and plays an important role in nonalcoholic fatty liver disease [86]. DLD may serve as a therapeutic target for energy metabolism in AD [87]. Together, the research of these genes reinforces the importance of mitochondria in AD. The results of this study may contribute to a better understanding of whether the interaction between mitochondrial gene signatures and immune cells influences AD progression.

This study is the first to identify the relationship between mitochondrial related genes and immune microenvironment in AD through bioinformatics analysis. BDH1, TRAP1, OPA1, DLD and SPG7 have been tested well as potential molecular targets for the diagnosis and prediction of AD risk. The expression levels of the five hub MitoDEGs have also been verified in cell and animal experiments. Of course, our research also has some limitations: 1. The brain region of the AD model we selected for analysis is the frontal and temporal cortex, which has not been verified in multiple other brain regions; 2. The specific roles of mitochondria-related genes in immune cells and their involvement in AD require further high-quality animal experimental verification. The possible signaling pathways affected by these hub genes and their functions in immune cells are our next research directions.

## Conclusions

We identified DEGs between AD and ND by comprehensive bioinformatics analysis and further investigated the mitochondrial genes associated with AD, and elucidated the tight relationship between hub mitoDEGs and immune cells, as well as immune microenvironment and AD pathology. Five hub MitoDEGs (BDH1, TRAP1, OPA1, DLD and SPG7) were screened and validated, and their mRAN expression levels decreased in AD, although there was no significant difference in the expression levels of SPG7. Most importantly, BDH1, TRAP1, OPA1, DLD and SPG7 were negatively correlated with a variety of immune cells, suggesting that these hub MitoDEGs are co-regulatory molecules of immunity and metabolism in AD. We further verified the expression levels of five hub MitoDEGs through in vivo and in vitro experiments, and discovered that OPA1 overexpression could reduce mitochondrial damage and neuronal apoptosis. Together, these findings contributed to a better understanding of the etiology of AD and provided new perspectives for exploring potential diagnostic markers and treatment strategies.

## Supplementary Information

**Additional file 1: Table S1.** The VIF values of the models.

**Additional file 2: Fig. S1.** Principal component analysis (PCA) of the two datasets.

**Additional file 3: File S1.** The top eight activated and suppressed gene sets from C2/C5 gene sets of MSigDB.

**Additional file 4: File S2.** The expression levels of DEGs/MitoDEGs in GSE122063 and GSE132903.

**Additional file 5: Fig. S2.** The positive/negative associations between down-regulated/upregulated mitoDEGs/hub mitoDEGs and immune cells.

**Additional file 6: Fig. S3.** The expression levels of hub mitoDEGs were verified in GSE132903, GSE44770 and GSE33000.

**Additional file 7: File S3.** The details of the hub MitoDEGs and clinical feature in GSE122063.

## Data Availability

All the data are freely available in the NCBI GEO, MitoCarta3.0, STRING and MSigDB databases. The experimental datasets used and/or analysed in the current study are available from the corresponding author on reasonable request. NCBI GEO, http://www.ncbi.nlm.nih.gov/geo; MitoCarta3.0, http://www.broadinstitute.org/mitocarta; MSigDB, https://www.gsea-msigdb.org/gsea/msigdb; STRING, https://string-db.org/.

## Abbreviations

AD: Alzheimer's disease
PD: Parkinson's disease
GEO: Gene Expression Omnibus
DEGs: Differential expression genes
APOE ε4: Apolipoprotein E ε4
Aβ: Amyloid β
p-tau: Phosphorylated tau
cGAS: Cyclic GMP-AMP synthase
STING: Stimulator of interferon genes
mtDNA: Mitochondrial DNA
PGE2: Prostaglandin E2
ND: Non-demented
MitoDEGs: Mitochondria‑related DEGs
GSEA: Gene set enrichment analysis
MSigDB: Molecular Signatures Database
LASSO: Least Absolute shrinkage and selection operator
SVM-RFE: Support vector machine-recursive feature elimination
ssGSEA: Single-sample gene-set enrichment analysis
PPI: Protein–protein interactions
VIF: Variance inflation factor
AUC: Area under the ROC curve
OXPHOS: Oxidative phosphorylation
ROS: Reactive oxygen species
Drp1: Dynamin-related protein 1
BDH1: β-Hydroxybutyrate dehydrogenase
AcAc: Acetoacetic acid
BHB: β-Hydroxybutyrate
TRAP1: Tumor necrosis factor receptor-associated protein 1
OPA1: Optic atrophy 1
SPG7: Spastic paraplegia type 7
DLD: Dihydrolipoamide dehydrogenase
NIT2: Nitrilase family member 2
MRP: Mitoribosomal protein
XPNPEP3: X-prolyl-aminopeptidase 3
SERHL2: Serine hydrolase-like2
TDRKH: Tudor and KH domain-containing
PEX11B: The peroxisomal membrane protein
NK: Natural killer
BBB: Blood–brain barrier
TCR: Receptors on T cells
TLRs: Toll-like receptors
