# Supplementary material for: Identification of mitochondrial related signature associated with immune microenvironment in Alzheimer’s disease
Source: J Transl Med. 2023 Jul 11;21:458. doi: 10.1186/s12967-023-04254-9 (PMC10334674; doi:10.1186/s12967-023-04254-9)
Supplement: Supplementary file 1 — Additional file 1: Table S1. The VIF values of the models. [file 12967_2023_4254_MOESM1_ESM.docx]

　　 Table S1 The VIF values of the models.

| The VIF values of the models | | | | | | |
| --- | --- | --- | --- | --- | --- | --- |
| sex | age | BDH1 | DLD | OPA1 | SPG7 | TRAP1 |
| 1.115 | 1.439362 | 2.015727 | 1.380278 | 1.21435 | 1.955457 | 1.624579 |
|  |  |  |  |  |  |  |
