# Supplementary figures and images for: Identification of mitochondrial related signature associated with immune microenvironment in Alzheimer’s disease
Source: J Transl Med. 2023 Jul 11;21:458. doi: 10.1186/s12967-023-04254-9 (PMC10334674; doi:10.1186/s12967-023-04254-9)

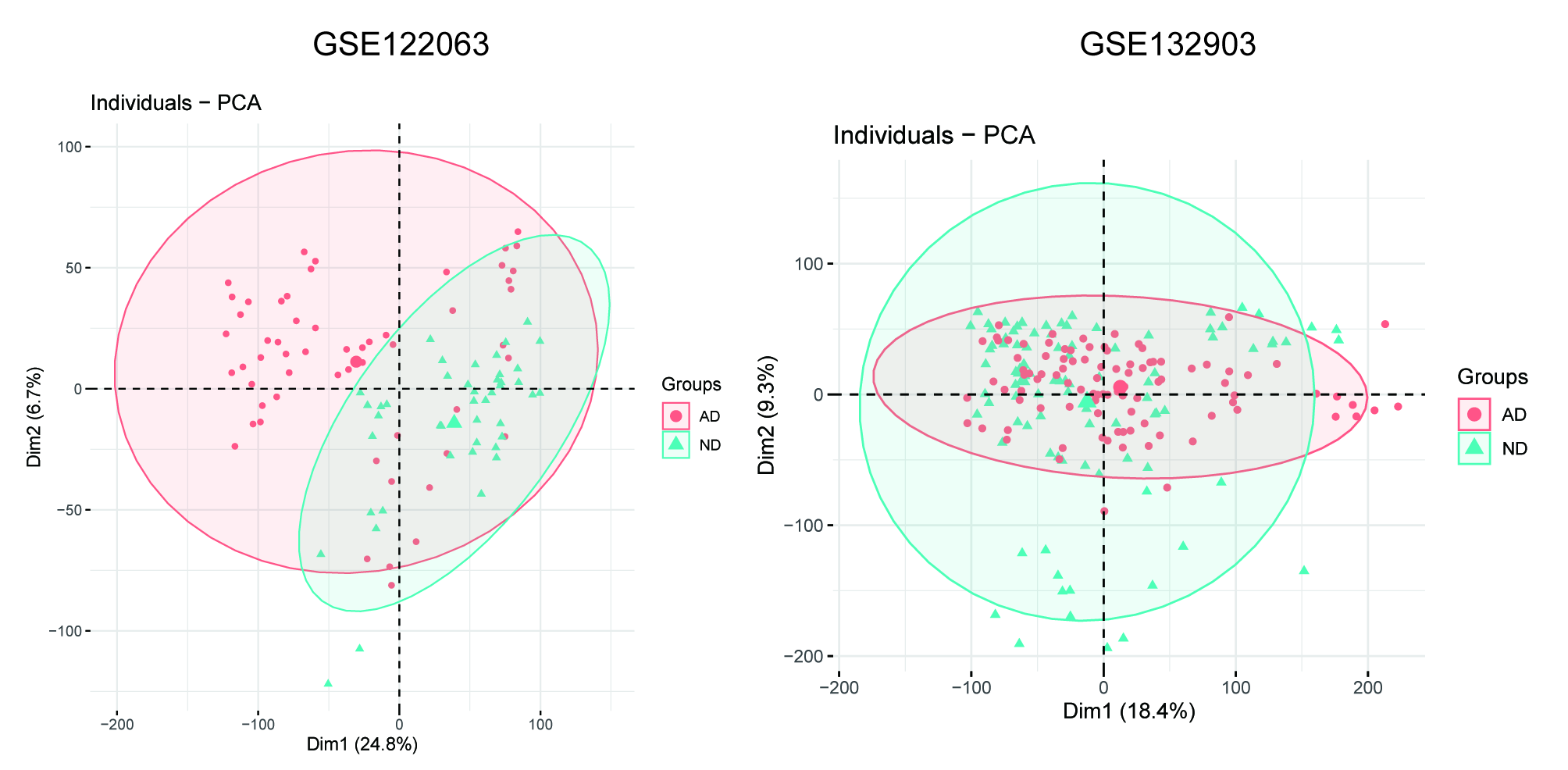

Supplement: Supplementary file 2 — Additional file 2: Fig. S1. Principal component analysis (PCA) of the two datasets. [file 12967_2023_4254_MOESM2_ESM.tif]

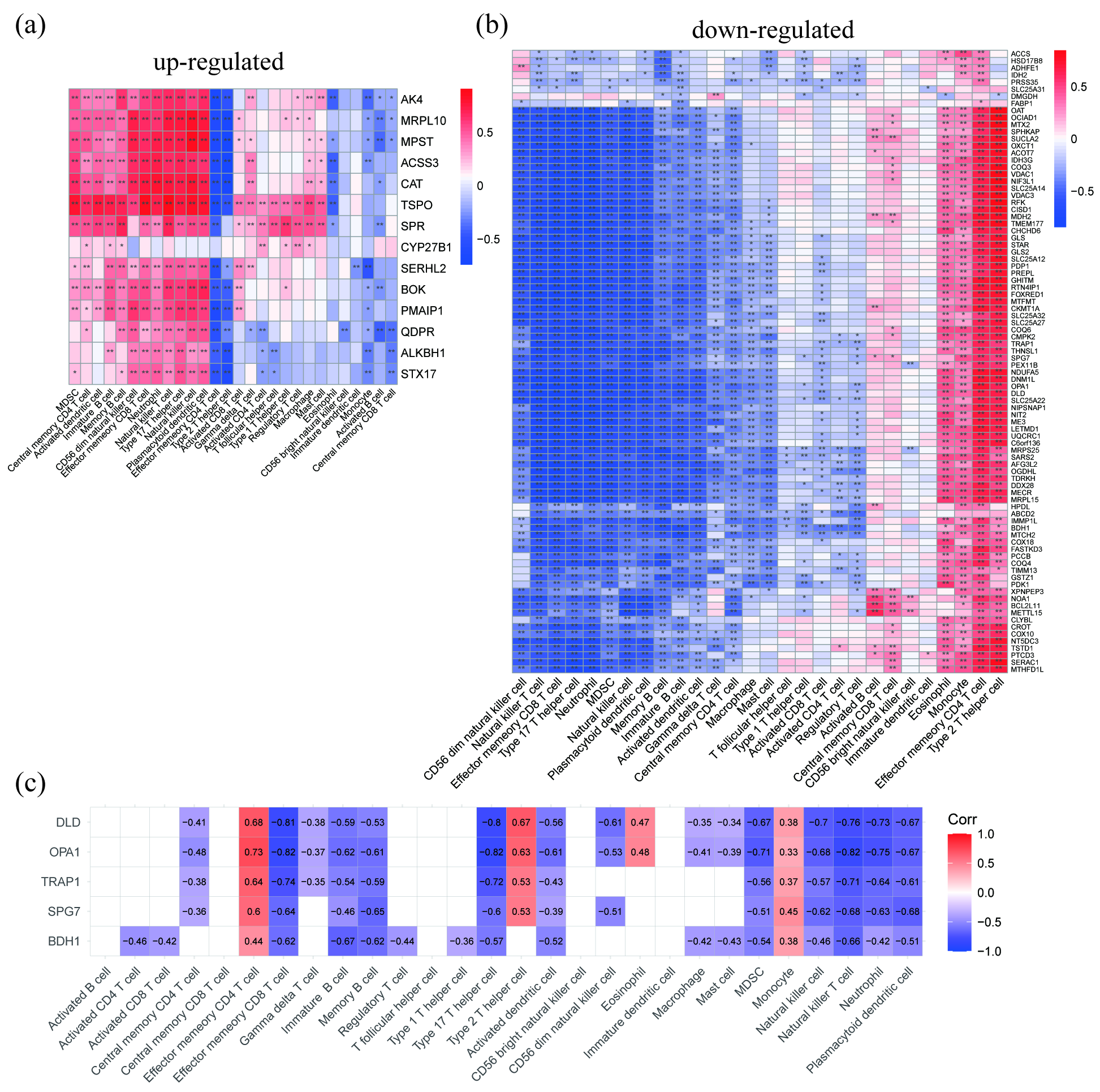

Supplement: Supplementary file 5 — Additional file 5: Fig. S2. The positive/negative associations between down-regulated/upregulated mitoDEGs/hub mitoDEGs and immune cells. [file 12967_2023_4254_MOESM5_ESM.tif]

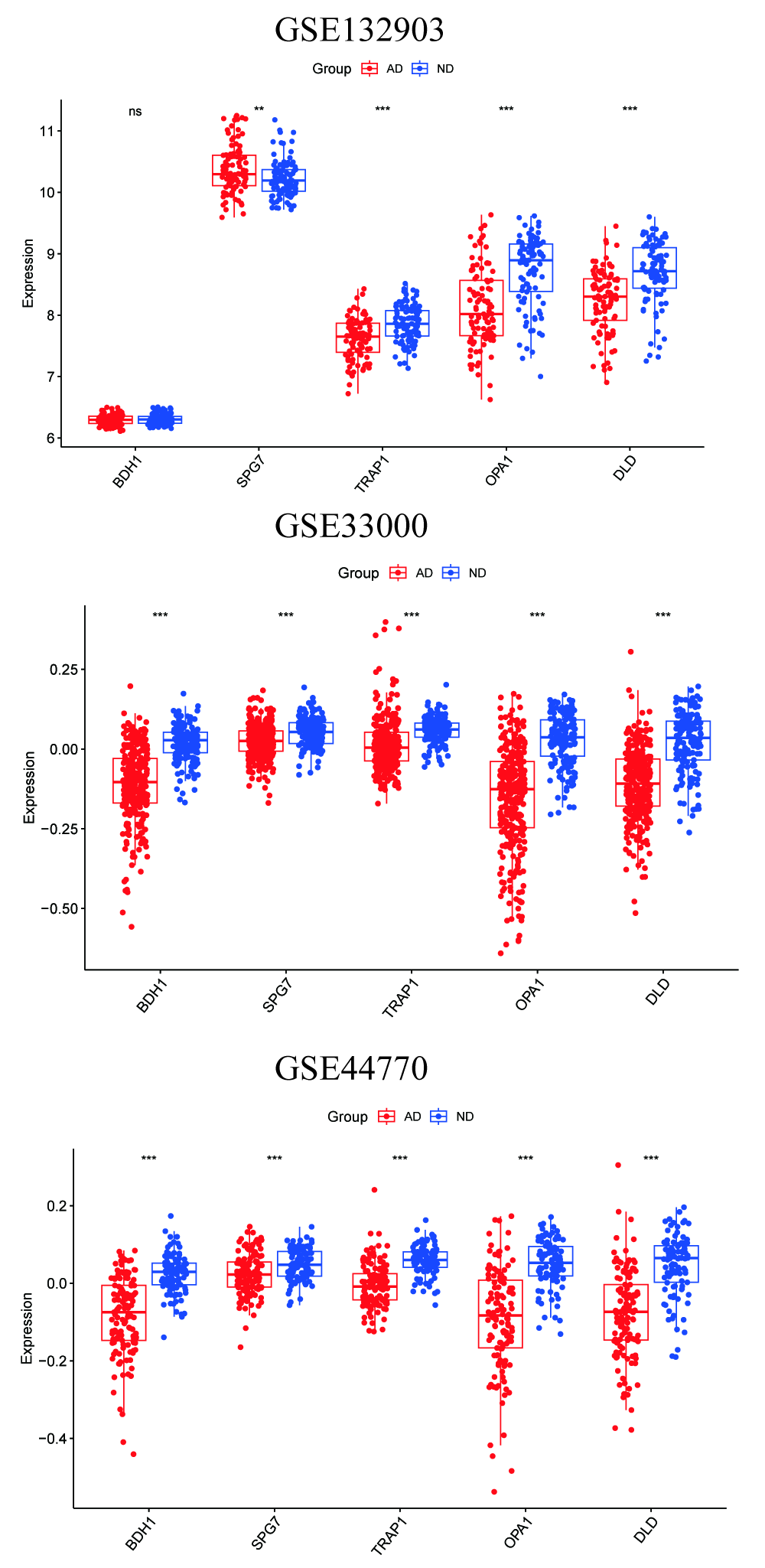

Supplement: Supplementary file 6 — Additional file 6: Fig. S3. The expression levels of hub mitoDEGs were verified in GSE132903, GSE44770 and GSE33000. [file 12967_2023_4254_MOESM6_ESM.tif]
